# Paediatric Posttonsillectomy Haemorrhage Rates in Auckland: A Retrospective Case Series

**DOI:** 10.1155/2019/4101034

**Published:** 2019-03-06

**Authors:** Andrés Alvo, Andrew Hall, James Johnston, Murali Mahadevan

**Affiliations:** ^1^Department of Paediatric Otorhinolaryngology, Starship Children's Hospital, Auckland, New Zealand; ^2^Department of Surgery, The University of Auckland, Auckland, New Zealand

## Abstract

**Background:**

Tonsillectomy is one of the most commonly performed surgical procedures in children. It is performed for sleep-disordered breathing and infective symptoms. Despite advances in instrumentation and perioperative care, posttonsillectomy haemorrhage (PTH) remains a significant complication, which should be audited regularly.

**Methods:**

A retrospective case series of all tonsillectomies performed in patients <15 years old in the Auckland region during 2017 was performed. Demographic, clinical, and surgical data were extracted and analysed. Univariate analysis was performed to compare patients with and without PTH.

**Results:**

A total of 2177 tonsillectomies were performed during the study period, 64% in a public hospital and 36% in a private hospital. The overall PTH rate was 3.6% (0.23% occurring within the first 24 hours (primary) and 3.4% after 24 hours (secondary)). Mean time to PTH was 6.6 ± 3 days (range: 1-16 days). 90% of PTH occurred within the first 10 days and 99% by 14 days. Return to theatre was required in 28% of these cases, representing 1% of all tonsillectomies. There were no deaths or major complications in this cohort. The only differences observed between patients with PTH and those without were that children with PTH had smaller tonsils (*p*=0.004) and were less likely to have associated OME (*p*<0.001).

**Conclusion:**

It is important to report institutional tonsillectomy outcomes and associated complications. These results show that PTH rates in Auckland remain within acceptable limits according to the literature.

## 1. Introduction

Tonsillectomy is one of the most commonly performed surgical procedures in the paediatric population worldwide [1]. 530,000 children under the age of 15 years undergo tonsillectomy each year in the United States alone [2]. Children usually undergo tonsillectomy as a consequence of tonsillar hyperplasia or recurrent infection. This can result in a broad spectrum of symptoms. At one end of the spectrum, children have obstructive sleep apnoea (OSA) and associated symptoms such as daytime sleepiness and fatigue, or hyperactivity and behavioural problems. At the other end there are children who complain of recurrent infective symptoms such as pain, fever, halitosis, and lymphadenopathy. Between these two poles there are children with a combination of both sleep-disordered breathing and infective symptoms [1]. Over the past several decades, sleep disordered breathing has become a much more common indication for tonsillectomy than recurrent tonsillitis [1]. This is important as children with sleep disordered breathing symptoms who undergo tonsillectomy have a reported increased risk of postoperative hemorrhage and need for postoperative readmission to hospital [1, 3].

Despite significant advances in surgical instrumentation and perioperative care, posttonsillectomy haemorrhage (PTH) remains a significant complication associated with tonsillectomy [4–6]. Clinical practice guidelines recommend institutions performing tonsillectomy undertake annual audit to ensure that PTH rates align with internationally published figures. It is also pertinent to review surgical technique and perioperative medications to identify any factors that may be associated with an increased risk of PTH [7]. Our institution in the Auckland region is unique in that all paediatric patients with PTH are reviewed and managed only at Starship Children's Hospital. This is irrespective of whether their operation was performed at a publicly or privately funded facility. Given this unique situation we can confidently generate an accurate number of cases occurring in this region. This retrospective case series aimed to determine Auckland regional PTH rates and associated demographic, clinical, and surgical characteristics. In addition, we aimed to compare children who underwent tonsillectomy with PTH versus those who did not, in order to determine any associated characteristics.

## 2. Methods

Data were obtained from Auckland District Health Board (ADHB), Counties Manukau District Health Board (CMDHB), and private hospitals in the Auckland region following national ethics committee approval (17/NTA/148). Extraction of all clinical information was performed following the retrospective identification of all patients under the age of 15 years undergoing tonsillectomy between the 1^st^ of January and the 31^st^ of December 2017.

The following demographic data were collected, as previously described by Johnston et al. [1]: age, sex, BMI, and ethnicity. Clinical data collection included the indication for surgery, tonsil grade, the presence of nasal symptoms, the presence of ear disease, medical comorbidities, antibiotics prescribed in the community prior to operation, and throat swab before admission. All medications prescribed upon discharge were reviewed and noted. Outcome data included postoperative visits to the general practitioner (GP), 30-day readmission rate, and associated complications including post-tonsillectomy bleeding. All demographic data were available for patients operated in the ADHB and CMDHB hospitals. We excluded 2 patients with PTH who had their primary surgery outside Auckland and 2 patients over 15 years of age that were admitted at Starship because of medical comorbidities warranting prolonged paediatric care.

Patient demographics and clinical characteristics were summarised using descriptive statistics. Univariate analysis was used to assess potential factors that were associated with differences in patients with PTH versus those without this postoperative complication.* Chi*-square tests were performed to assess categorical variables and the ANOVA test was conducted to evaluate continuous variables. A two-tailed* p*-value < 0.05 was regarded as statistically significant. IBM® SPSS® version 24 software was used to perform all statistical analyses.

## 3. Results

From January 1 to December 31 of 2017, a total of 2177 tonsillectomies were performed on paediatric patients in the Auckland Region. Tonsillectomy alone was performed in 15% (n=326) of cases, whereas all other surgeries included at least one additional procedure including adenoidectomy and inferior turbinate cautery. 64% (n=1395) of tonsillectomies were performed in a publicly funded hospital, with the remainder (36%, n=782) being performed in a private hospital.

The rate of PTH in this cohort was 3.6% (n=78). 2.6% of all PTH patients had more than one episode (n=2/78). In the public hospital cohort, PTH occurred in 3.9% (n=54/1395) of patients, compared with 3.1% (n=24/782) in the private hospital group (*p* = 0.33). Mean patient age was 7.5 ± 3.5 years (range 1–15 years). 51% (n=38/78) of PTH patients were male.

**Figure 1:**
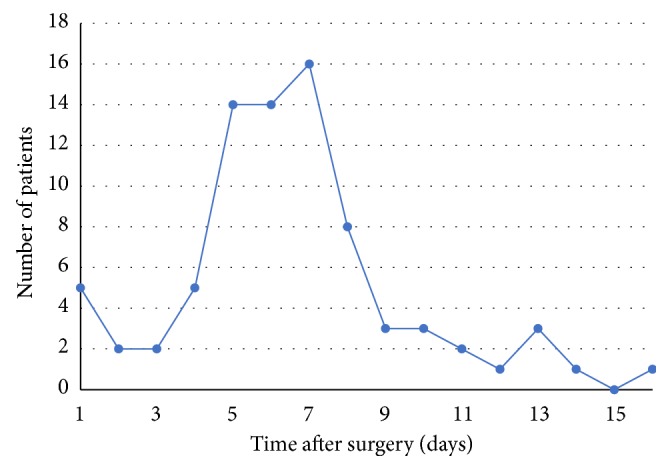
Incidence of posttonsillectomy haemorrhage according to time after surgery.

Mean time to PTH from the day of surgery was 6.6 ± 3 days (range 1 – 16 days). PTH occurring within 24 hours of surgery is regarded as primary PTH and occurred in 0.23% (n=5/2177), accounting for 6% (n=5/78) of all patients with PTH. One of these patients had a secondary PTH on postoperative day 16. Secondary PTH occurred in 3.4% of all tonsillectomies (73/2177); one of these patients had two episodes, on days 4 and 6. 90% of all PTH occurred within the first 10 days (n=72/80) and 99% in the first 14 days (n=79/80). The day of presentation to hospital with PTH in all patients is shown in Figure 1.

Indications for tonsillectomy in patients with PTH included sleep-disordered breathing in 54% (n=42/78), recurrent tonsillitis in 24% (n=19/78) and a combination of indications in 6% (n=5/78). In the remaining 16% (n=12/78) of patients this information was unavailable. In regard to surgical technique, cold steel dissection with bipolar diathermy was used in 36% (n=28/78) of cases, bipolar diathermy only in 37% (n=29/78) of cases, monopolar diathermy in 10% (n=8/78) of cases, and coblation in 5% (n=4/78) of cases. In the remaining 12% (n=9/78) of cases information about the surgical technique was not available. Non-steroidal anti-inflammatory drugs (NSAIDs) were prescribed to 82% (n=64/78) of patients with PTH.

In regard to the management of patients with PTH, 28% (n=22/80) had a general anaesthetic and arrest of haemorrhage performed in theatre (1.01% of all tonsillectomies); the remaining 72% (n=58/80) were managed conservatively. In primary PTH, 40% returned to theatre (n=2/5) while in secondary PTH 27.4% did (n=20/73). There are 80 episodes of PTH included in this analysis given that two patients had a second PTH episode. Both of these patients were managed conservatively. The mean haemoglobin level on admission was 121.4 g/L (range from 64 g/L to 162 g/L). Only one patient required a blood transfusion, as haemoglobin had dropped to 64 g/L. There were no deaths related to PTH in this cohort.

A comparison of clinical and demographic factors was undertaken in patients who had PTH versus those who did not. Only the 1395 patients who were operated on in publicly funded hospitals were included in this analysis given the paucity of data available in those patients operated on in the private sector. There were 54 patients who had a PTH and 1341 who did not. Demographic and clinical characteristics are summarised in Table 1.

Patients in the PTH group had smaller tonsils than those in the non-PTH group (PTH 2.8 ± 0.2 and no PTH 3.1 ± 0.03;* p*=0.004). There was no significant difference in adenoid grade between groups (PTH 2.8 ± 0.2 and no PTH 2.6 ± 0.05;* p*=0.06). No significant differences were noted in the presence of medical comorbidities including asthma, eczema, allergic rhinitis, and gastroesophageal reflux disease (GORD) (*p*=0.18,* p*=0.25,* p*=0.52, and* p*=0.07, respectively).

Children with PTH were less likely to have associated otitis media with effusion requiring ventilation tube insertion at the time of surgery (1.9% PTH, compared with 25.1% no PTH;* p*<0.001). There was no statistical difference in the incidence of a documented history of speech delay between groups (13% PTH, compared with 17.5% no PTH;* p*=0.39).

A throat swab for Group-A Streptococcus (GAS) was performed in 75.9% of children with PTH and 77.1% with no PTH in the year preceding surgery (*p*=0.84). These swab results were positive for GAS in 27.8% of children with PTH and 35.2% with no PTH in the year preceding surgery (*p*=0.26). There was no difference between groups in regard to medications prescribed at the time of discharge including antibiotics, paracetamol, ibuprofen, and tramadol (*p*=0.83,* p*=0.23,* p*=0.81, and* p*=0.71, respectively).

## 4. Discussion

Tonsillectomy continues to be one of the most commonly performed procedures worldwide. However, there is significant variability in clinical practice, lack of strong evidence-based guidelines, and scarce availability of quality registers [8, 9].

Our records show Auckland's PTH rates to be consistent with those reported in the literature [5, 10–12]. There were no significant differences observed between public and private patients. We had a low incidence of primary PTH and, as expected, the highest number of PTH occurred between postoperative days 5 and 7. 90% of PTH occurred during the first 10 postoperative days and 99% within 14 days. During the studied period, we had no PTH beyond day 16.

Approximately 25% of patients with PTH who were admitted required a return to theatre to arrest bleeding. In other words, 1% of paediatric tonsillectomy patients require a second general anaesthetic and surgical procedure. This finding is consistent with other studies [13]. This is a significant complication of tonsillectomy and patients should be informed of this prior to surgery.

In this cohort there were no cases of major haemorrhage requiring external carotid ligation or embolization, or resulting in death. While these outcomes are extremely infrequent, they are well described in the literature [14, 15]. PTH is regarded as the main cause of malpractice claims in paediatric tonsil surgery in the United States of America [16].

The only differences observed between patients with PTH and those without were that children with PTH had smaller tonsils at the time of surgery and were less likely to have associated OME. To our knowledge this finding has not previously been reported. Otherwise there were no observed differences in clinical, demographic, or surgical characteristics between the two groups of patients.

Two of the main challenges of reporting PTH rates and associated factors are the lack of a universally-accepted classification system and the various ways of recognising and reporting episodes. In regard to the first challenge, several studies have attempted to standardise bleeding severity, mainly based on the need for admission, interventions, or outcomes [17, 18]; but still these criteria are greatly subjective and practice patterns can vary widely between surgeons or institutions [19, 20]. In our institution, all PTH presenting to the Emergency Department are admitted for overnight observation and medical management. Return to theatre is decided if there is active bleeding, clot growing or persisting after observation, drop in haemoglobin levels below 100 g/L, blood loss greater than 10 mL/kg, or hypovolaemia. This is in line with commonly accepted management strategies, as reported by a recent survey on members of the American Society of Pediatric Otolaryngology [19].

In regard to the second challenge, different institutions may have varying definitions on what constitutes PTH. This may range from a small amount of blood-stained saliva to active bleeding requiring admission or surgery [17, 21]. Reported rates of PTH will therefore vary depending on the definition used. As this is a retrospective study, we included only PTH requiring admission to hospital for either surgical intervention or conservative management.

An audit conducted in Dunedin, New Zealand, reported an increase in PTH (including children and adults), finding a correlation with newer surgical instruments (such as diathermy and coblation), and a change in the use of routine drugs (NSAIDs and steroids) [22], while another audit performed in the United Kingdom identified a significant improvement of PTH rates in children and adults after the use of coblation was abandoned [23].

In the majority of our PTH patients, sleep-disordered breathing was the most frequent indication for tonsil surgery, which is consistent with the shift from infectious to obstructive indications observed worldwide [24, 25]. Although in New Zealand extracapsular tonsillectomy is still the surgical standard for obstructive symptoms associated with tonsillar hyperplasia in children, there is growing evidence showing that tonsillotomy (intracapsular or partial tonsillectomy) could provide similar results, with less postoperative pain and lower bleeding rates [26, 27]. The effect of choosing tonsillotomy on PTH rates in our setting is yet to be determined and could be a matter of further studies.

Retrospective studies, such as this, rely upon accurate clinical records. Therefore, only PTH severe enough to warrant admission to hospital would be registered and included. It is possible that the rate of PTH may be significantly underreported. In fact, one study showed that up to one-fifth of all PTH episodes could be missed by this approach [28], especially in cases where the bleeding spontaneously stops and patients do not present, or if they are seen by a doctor in the community and not referred to hospital. For all of these reasons, comparison of PTH rates between studies is extremely difficult.

## 5. Conclusion

It is important to report institutional tonsillectomy outcomes and associated complications. Our report highlights the current status of paediatric PTH in Auckland over a 1-year period and its demographic characteristics, showing no major changes with previous reports and remaining within acceptable limits according to the literature.

## Figures and Tables

**Table 1:** Demographic and preoperative prescription data for children who underwent tonsillectomy in the public sector: PTH versus no PTH.

	PTH (n=54)	No PTH (n=1341)	*p*-value
*Gender*			

Male	53.7%	57.5%	*p*=0.58

Female	46.3%	42.5%	*p*=0.58

*Mean age (years)*	6.5 ± 0.9	6.9 ± 0.2	*p*=0.28

*Mean Body Mass Index*	20.4 ± 1.7	19.3 ± 0.3	*p*=0.1

*Courses of antibiotics in the year before surgery*	3.3 ± 0.4	3.4 ± 0.1	*p*=0.74

*Analgesia prescribed by a general practitioner in the year before surgery*	72.2%	80.3%	*p*=0.15

*Prednisone prescribed by a general practitioner in the year before surgery*	14.8%	17.7%	*p*=0.58

†PTH = Posttonsillectomy haemorrhage.

## Data Availability

The data used to support the findings of this study are included within the article.

## References

[1] Johnston J., McLaren H., Mahadevan M., Douglas R. G. (2019). Clinical characteristics of obstructive sleep apnea versus infectious adenotonsillar hyperplasia in children. *International Journal of Pediatric Otorhinolaryngology*.

[2] Cullen K. A., Hall M. J., Golosinskiy A. (2009). Ambulatory surgery in the United States. *Natl Health Stat Report*.

[3] Sunnergren O., Odhagen E., Stalfors J. (2017). Incidence of second surgery following pediatric adenotonsillar surgery: a population-based cohort study. *European Archives of Oto-Rhino-Laryngology*.

[4] Johnson L. B., Elluru R. G., Myer C. M. (2002). Complications of Adenotonsillectomy. *The Laryngoscope*.

[5] Francis D. O., Fonnesbeck C., Sathe N., McPheeters M., Krishnaswami S., Chinnadurai S. (2017). Postoperative bleeding and associated utilization following tonsillectomy in children: a systematic review and meta-analysis. *Otolaryngology–Head and Neck Surgery*.

[6] Acevedo J. L., Shah R. K., Brietzke S. E. (2012). Systematic Review of Complications of Tonsillotomy versus Tonsillectomy. *Otolaryngology—Head and Neck Surgery*.

[7] Baugh R. F., Archer S. M., Mitchell R. B. (2011). Clinical Practice Guideline: Tonsillectomy in Children. *Otolaryngology—Head and Neck Surgery*.

[8] Ruohoalho J., Østvoll E., Bratt M. (2018). Systematic review of tonsil surgery quality registers and introduction of the Nordic Tonsil Surgery Register Collaboration. *European Archives of Oto-Rhino-Laryngology*.

[9] Venekamp R. P., Hearne B. J., Chandrasekharan D., Blackshaw H., Lim J., Schilder A. G. (2015). Tonsillectomy or adenotonsillectomy versus non-surgical management for obstructive sleep-disordered breathing in children. *Cochrane Database of Systematic Reviews*.

[10] Mahadevan M., van der Meer G., Gruber M. (2016). The starship children's hospital tonsillectomy: A further 10 years of experience. *The Laryngoscope*.

[11] Krishna P., Lee D. (2001). Post-Tonsillectomy Bleeding: A Meta-Analysis. *The Laryngoscope*.

[12] Wall J. J., Tay K. (2018). Postoperative Tonsillectomy Hemorrhage. *Emergency Medicine Clinics of North America*.

[13] Reusser N. M., Bender R. W., Agrawal N. A., Albright J. T., Duncan N. O., Edmonds J. L. (2017). Post-tonsillectomy hemorrhage rates in children compared by surgical technique. *Ent-Ear Nose & Throat Journal*.

[14] Østvoll E., Sunnergren O., Ericsson E. (2015). Mortality after tonsil surgery, a population study, covering eight years and 82,527 operations in Sweden. *European Archives of Oto-Rhino-Laryngology*.

[15] Windfuhr J. P. (2013). Serious Complications following Tonsillectomy: How Frequent Are They Really?. *ORL*.

[16] Subramanyam R., Varughese A., Willging J. P., Sadhasivam S. (2013). Future of pediatric tonsillectomy and perioperative outcomes. *International Journal of Pediatric Otorhinolaryngology*.

[17] Walner D. L., Karas A. (2013). Standardization of Reporting Post-Tonsillectomy Bleeding. *Annals of Otology, Rhinology & Laryngology*.

[18] Windfuhr J., Seehafer M. (2001). Classification of haemorrhage following tonsillectomy. *The Journal of Laryngology & Otology*.

[19] El Rassi E., de Alarcon A., Lam D. (2017). Practice patterns in the management of post-tonsillectomy hemorrhage: An American Society of Pediatric Otolaryngology survey. *International Journal of Pediatric Otorhinolaryngology*.

[20] Clark C. M., Schubart J. R., Carr M. M. (2018). Trends in the management of secondary post-tonsillectomy hemorrhage in children. *International Journal of Pediatric Otorhinolaryngology*.

[21] Whelan R. L., Shaffer A., Anderson M. E., Hsu J., Jabbour N. (2018). Reducing rates of operative intervention for pediatric post-tonsillectomy hemorrhage. *The Laryngoscope*.

[22] Macassey E. A., Baguley C., Dawes P., Gray A. (2007). 15-year audit of post-tonsillectomy haemorrhage at dunedin hospital. *ANZ Journal of Surgery*.

[23] Javed F., Sadri >., Uddin J., Mortimore S., Parker D. (2009). A completed audit cycle on post-tonsillectomy haemorrhage rate: Coblation versus standard tonsillectomy. *Acta Oto-Laryngologica*.

[24] Erickson B. K., Larson D. R., St. Sauver J. L., Meverden R. A., Orvidas L. J. (2009). Changes in incidence and indications of tonsillectomy and adenotonsillectomy, 1970–2005. *Otolaryngology—Head and Neck Surgery*.

[25] Parker N. P., Walner D. L. (2011). Trends in the indications for pediatric tonsillectomy or adenotonsillectomy. *International Journal of Pediatric Otorhinolaryngology*.

[26] Kim J. S., Kwon S. H., Lee E. J., Yoon Y. J. (2017). Can intracapsular tonsillectomy be an alternative to classical tonsillectomy? A meta-analysis. *Otolaryngology—Head and Neck Surgery*.

[27] Zhang L., Zhong L., David M., Cervin A. (2017). Tonsillectomy or tonsillotomy? A systematic review for paediatric sleep-disordered breathing. *International Journal of Pediatric Otorhinolaryngology*.

[28] Sarny S., Habermann W., Ossimitz G., Schmid C., Stammberger H. (2011). Tonsilar haemorrhage and re-admission: a questionnaire based study. *European Archives of Oto-Rhino-Laryngology*.

